# Unification: A Fatal Error

**Published:** 1882-01

**Authors:** Ad. Lippe

**Affiliations:** Phila.


### UNIFICATION: A FATAL ERROR.

AD. LIPPE, M. D., PHILA.

IT is a fatal error to suppose that partition walls, separating the medical schools are to come down. Following the inductive method of Hahnemann, we find him from the outset exposing the follies, the the varying hypotheses, and the want of success of the then prevailing school of medicine. This foundation for his subsequent argument was a logical necessity; and the argument following these declarative exposures of the condition of the prevailing school of medicine did build up an insurmountable partition wall, separating forever the Healing-Art, promulgated by Samuel Hahnemann, including his exclusive therapeutics, from the prevailing medical school. The partition walls can surely not come down till some very learned chemist has discovered the method of mixing oil and water. Any person, comprehending the absolute and irreconcilable differences existing between the two schools, can no more dream of the possibility of uniting them, than can the modern chemist see the possibility of uniting oil and water. As these differing schools cannot unite, the taking down of the partition wall implies a "surrender" of one side or the other; this also seems very problematical, as progressive homœopathists, who are a goodly number, get still further away and out of the baneful influence of the prevailing school of medicine. Some members of the ordinary school are already innocently adopting the various doctrines taught by Hahnemann. It is a logical necessity that in the course of time, in the very dim future, these materialists, who shun light and progress, now must leave their dearly-beloved, conservative but untenable ground. Thus, gradually, without any aid from our side, they will remove by their own strong exertions, portion after portion of that partition wall; and as they do so, more light will strike them, and the more they are illumined by the ever-increasing light, the faster will progress the level ling work until there shall be no partition wall any longer. The signs of the times disclose the fact that an occasional glimpse of light reaches over the partition wall, yet is evident that the conservatism of centuries still pervades the common school of medicine. They are, have been, and will for some time continue to be, conservative in their materialism; the progress they make being very slow, and under pressure of public opinion. To make good our argument we must show the position the common school of medicine holds to-day, and illustrate their persistent conservatism. Prof. Pasteur read before a Medical Congress, assembled at London during last summer, his famous paper on "Preventive Medicine." The famous Pasteur is a "*chemist*," and as such, deals exclusively with organic matter; his observations are of the greatest interest to every scientific man. Pasteur addresses the allopathic physicians, who are still engaged in searching out the material causes of diseases; they full well understand that the homœopathists accept with the law of the similars also the law of dynamics, as two inseparable and homogeneous doctrines, promulgated by Hahnemann and accepted by his followers,* rejected, of course, by the old school still adhering to materialism. Pasteur claims to have laid the foundation of an entirely new system and method of research into the nature and causes of a large class of diseases in man and the higher animals; he proclaims that a large class of diseases is curable, under the maxim, "*æqualia æqualibus curantur*," and he, not adhering to a strict inductive method of reasoning, bases his experiment on the discovery of the immortal Jenner; but Jenner vaccinated mankind with a "*similar*" virus, obtained from a lower animal, and therefore—it being only similar—he prevented men from being afflicted by small-pox. Jenner did not claim that the lymph taken from a small-pox patient would either prevent or cure the disease in other persons. The profession at large could not possibly accept Pasteur's newly-discovered law of cure, because they were observing and practical men. From times immemorial have men of undoubted learning vainly searched for specific remedies for specific diseases. It could not be otherwise as their very first proposition, the existence of specific diseases, is a fatal error, an error first and last. From times immemorial diseases have continually changed their nature and forms. What at present appears to correspond with

---

* *London Lancet*, June, 1881, page 553.

the *genus epidemicus*, the various phenomena which appear to be strongly expressed in all forms of the now prevailing disease, will no longer be characteristic accompaniments of this same disease, probably three months hence. From times immemorial persons suffering at the same place and at the same time from epidemic diseases were all afflicted similarly, but not alike; while there existed an apparent great similarity between the afflicted, the close observer readily discerned a great difference between the symptoms of the similarly-sick. These close observers were Hahnemann and his disciples, and as illustration we may be allowed to refer to some very frequently indicated remedies in the Asiatic Cholera. While some cases corresponded with the characteristic sick-making properties (ascertained from provings on the healthy) of Camphor, other cases corresponded with Veratrum or Cuprum or Arsenic, etc. Each of these remedies had its characteristics, and became thereby and therefore a curative agent under the law of the similars. Pasteur, as well as Koch and others, profess to have found the germs of infectious diseases, and believing that they have found these germs, they come to the conclusion, following their deductive method of reasoning, that they also know how to stamp out these diseases. When, heretofore, an infectious disease broke out in a certain locality, there were necessarily several conditions present, allowing the germs to to develop themselves and their infectious character; after a certain time the germs, now having rapidly multiplied, were found to become harmless, as persons long exposed to their influences remained well, having no susceptibility to that specific poison; then, what became of these multiplied germs? Can chemistry or any other exact science explain? No more than they can explain why A, B and C became ill when exposed to these poisonous germs, and why D, E and F were not at all affected by the same influences. If, after a long lapse of time, an epidemic breaks out where it formerly raged, is not that new epidemic invariably very different from the former one? If we are accustomed to individualize, it is hardly to be expected that we should even think of accepting such positive generalizations as are offered us by these scientists, certainly we should not accept them as therapeutic guides. The allopathists have not accepted as a body, nor any considerable number of them, the newly-discovered law of cure promulgated by Pasteur—that the product of a disease would cure the disease itself, or if others were inoculated with this product, would serve as a preventive for that form of a disease. This formula, *æqualia æqualibus curantur*, was rejected; and how could it be otherwise? Pasteur is a scientist, but not a practical man, nor was he the first man who offered this formula.

More than fifty years ago Mr. M. Lux published a pamphlet, declaring the law, *æqualia æqualibus curantur*, superior to the law of the similars. Mr. Lux was a veterinary surgeon. Men of that profession were not compelled to show themselves to be graduates of a classical college like the matriculants in the universities, who enter upon the study of medicine. It could, therefore, not be supposed that Mr. Lux would take in the inductive method of Hahnemann, and fully understand and appreciate his Organon, when so many, nay, the great majority of well-educated medical men rejected it *a priori*, because they were unwilling to try the experiment. Mr. Lux was an enthusiast; a few followed him, and among them, Dr. Gross, a true homœopath. There was a prospective partition wall to be erected between the homœopathists and isopathists, but in his wisdom, Hahnemann made that utterly impossible, when he wrote a long foot-note at the close of his introduction to the 5th edition of his Organon. Dr. Gross saw his error and renounced it, and from that time till now, isopathy was remembered only as a medical folly.

It is a historical fact that Hahnemann, the founder of the Homœopathic Healing-Art, rejected isopathy in 1830; it is also a historical fact that isopathy was offered the allopathic school in 1881 by Prof. Pasteur, and that it was not accepted. It is also a historical fact that men who never fully adopted Homœopathy, and only pretended to belong to that school for reasons best known to themselves, but not to be publicly questioned, have declared from time to time a strong wish to have the partition wall, which separates the two great medical schools, thrown down. The supposition that such an event could take place has been discussed above, but a very ingenious medical journal* (ostensibly homœopathic) has, of late, made quite a smart proposition—he lets the wall stand and mounts it, holding in his hand an olive-branch, with the motto: "A scientific basis for Isopathy." This paper is written in a truly allopathic (materialistic) style. Individualization, the prerogative of true Homœopathy, is set aside entirely. Products of diseases (so-called) may and will become curative agents after they have been proved

* *United States Medical Investigator*, Nov. 1st and 15th, 1881.

on the healthy; may and will be applied for the cure of the sick under the homœopathic fundamental law of the Similars, the single remedy and the minimum dose applied, not singly, but jointly. The same decisive cures as we now get from treating the sick with the greatest known poisons, and with substances formerly considered inert, will follow. Whether the ingeniously offered olive-branch will be accepted by a small minority of dissatisfied allopathists, and if so accepted, whether a new medical school will be the outcome of this novel exhibition, a school advocating the principle, "*æqualia æqualibus curantur,*" and named isopathy, time alone will show. But that the partition wall will not be removed by this most novel peace-offering is a certainty.

